# Development of an artificial intelligence algorithm for automated surgical gestures annotation

**DOI:** 10.1007/s11701-025-02556-2

**Published:** 2025-07-18

**Authors:** Rikke Groth Olsen, Flemming Bjerrum, Annarita Ghosh Andersen, Lars Konge, Andreas Røder, Morten Bo Søndergaard Svendsen

**Affiliations:** 1 Department of Urology, Copenhagen Prostate Cancer Center, Copenhagen University Hospital-Rigshospitalet, Copenhagen, Denmark; 2 Copenhagen Academy for Medical Education and Simulation (CAMES), Centre for HR & Education, the Capital Region of Denmark, Ryesgade 53B, 2100 Copenhagen, Denmark; 3 Department of Clinical Medicine, Faculty of Health and Medical Sciences, University of Copenhagen, Copenhagen, Denmark; 4 Gastrounit, Surgical Section, Copenhagen University Hospital - Amager and Hvidovre, Hvidovre, Denmark; 5 Department of Cardiothoracic Surgery, Copenhagen University Hospital – Rigshospitalet, Copenhagen, Denmark; 6 Drug Delivery and Sensing (IDUN), Health Tech, Technical University of Denmark, Lyngby, Denmark

**Keywords:** Artificial intelligence, Surgical gestures, Video Assessment, Robot-assisted radical prostatectomy, Simulation

## Abstract

Surgical gestures analysis is a promising method to assess surgical procedure quality, but manual annotation is time-consuming. We aimed to develop a recurrent neural network for automated surgical gesture annotation using simulated robot-assisted radical prostatectomies. We have previously manually annotated 161 videos with five different surgical gestures (R*egular dissection, Hemostatic control, Clip application, Needle handling,* and *Suturing*). We created a model consisting of two neural networks: a pre-trained *feature extractor* (VisionTransformer using Imagenet) and a *classification head* (recurrent neural network with a Long Short-Term Memory (LSTM(128) and fully connected layer)). The data set was split into a training + validation set and a test set. The trained model labeled input sequences with one of the five surgical gestures. The overall performance of the neural networks was assessed by metrics for multi-label classification and defined *Total Agreement*, an extended version of Intersection over Union (IoU). Our neural network could predict the class of surgical gestures with an Area Under the Curve (AUC) of 0.95 (95% CI 0.93–0.96) and an F1-score of 0.71 (95% CI 0.67–0.75). The network could classify each surgical gesture with high accuracies (0.84–0.97) and high specificities (0.90–0.99), but with lower sensitivities (0.62–0.81). The average Total Agreement for each gesture class was between 0.72 (95% CI ± 0.03) and 0.91 (95% CI ± 0.02). We successfully developed a high-performing neural network to analyze gestures in simulated surgical procedures. Our next step is to use the network to annotate videos and evaluate their efficacy in predicting patient outcomes.

**Supplementary Information:**

The online version contains supplementary material available at 10.1007/s11701-025-02556-2.

## Introduction

Focus on the relevance and possibilities of artificial intelligence (AI) in health care has increased over the years [1]. AI is a field of computer science that uses networks to enable machines to learn from existing data and make predictions based on new, unseen data. This has the advantage of reducing human bias and improving decision-making when clinicians must process a massive amount of patient health information from various sources, like medical records, lab results, and robotic systems [2–5]. In recent years, AI has been used to predict patient outcomes in robot-assisted radical prostatectomies (RARP) by analyzing *surgical gestures* from surgical videos [6–8]. Surgical gestures are the surgeon's small actions during surgery, such as dissection, hemostatic control, and needle handling. [9, 10]. The surgical gestures are typically manually labeled by reviewing a recorded surgical video by a qualified person trained in video analysis. This annotation of surgical gestures onto the videos creates the unique surgical workflow patterns of each surgery needed for the AI networks to analyze. These AI networks could help guide treatment decisions. However, the annotation process is very time-consuming and not scalable to large data sets, which is needed to build robust networks [11].

Therefore, we wanted to develop a neural network for *automated* surgical gesture annotations. We created the network using previously annotated videos of simulated RARPs.

## Methods

### Data collection

In a previous study, we collected data on surgeons performing RARP on the RobotiX Mentor Simulator™ (Surgical Science, Gothenburg, Sweden) [12]. Ten novice surgeons with knowledge of the procedure (had assisted one or more RARP but never performed robotic surgery) and seven experienced RARP surgeons (performed ≥ 50 RARP) performed a maximum of three repetitions on each of the three part-procedures of the RARP: *Bladder-neck dissection (BND), Neurovascular-bundle dissection (NVBD),* and *Urethrovesical anastomosis (UVA)*. Full-length videos of each part of the procedure were automatically recorded on the simulator. All videos were manually annotated by the principal investigator RGO using the event-logging software *Behavioral Observation Research Interactive Software (*BORIS, version 8.19.4, Torino*,* Italy, http://www.boris.unito.it) [13]. The videos were annotated with five different surgical gestures: R*egular dissection, Hemostatic control, Clip application, Needle handling,* and *Suturing* (Table 1). Further details on the annotation process available in previous publications by Olsen et al. [10, 14].

**Table 1:** The neural network's classification performance for each class of surgical gestures showed high accuracy and specificity, with a lower performance in sensitivity

				Specificity	Sensitivity	**Accuracy**
	Gestures		Definition	*Mean (95% confidence interval)*		
Dissection	G1	Regular dissection	Any instrument performs either blunt or sharp dissection	0.91 (0.86–0.97)	0.73 (0.65–0.81)	0.87 (0.83–0.91)
	G2	Hemostatic control	Any instrument performs hemostatic control using monopolar or bipolar energy	0.94 (0.92–0.95)	0.75 (0.62–0.89)	0.89 (0.85–0.93)
	G3	Applications of clips	A clip is applied to the tissue	0.99 (0.98–0.99)	0.81 (0.71–0.92)	0.97 (0.95–0.98)
Suturing	G4	Needle handling	One of the needle handlers is in touch with the needle	0.91 (0.86–0.96)	0.62 (0.47–0.78)	0.85 (0.83–0.87)
	G5	Suturing	The needle is in touch with the tissue	0.90 (0.85–0.94)	0.68 (0.56–0.81)	0.84 (0.82–0.87)

### System design

A system was created to automatically annotate video sequences with the five different surgical gestures. The model consists of two neural networks in series: a transformer-based encoder network feeding to a recurrent neural network.

#### Processing, feature extraction, and sequence prediction

This model aimed to extract features from a sequence of frames in the video and, via training, predict one of the five classes of surgical gestures.

The model consisted of two individual networks: a pre-trained *feature extractor* (VisionTransformer using Imagenet) and a *classification head* on top*.* The classification head was the only network updated during the model training.

The feature extractor is an untrained vision transformer used to reduce the number of dimensions in the frames by extracting features from each frame. This makes the image easier to process for the network without losing important features. Different feature extractors were analyzed using the Monte-Carlo cross-validation to find the best-performing one (VIT, INception, Resnet) with a Vision Transformer trained on ‘ImageNet’ performing the best. ImageNet is a database where others have trained the network to assign a class to everyday objects, e.g., animals, furniture, persons, etc. The ImageNet does not contain surgical images but has been used as a basis for feature extractors for pathological images and surgical video analysis in previous studies [7, 15].

The *classification head* is a trained recurrent neural network with a Long Short-Term Memory (LSTM(128)) architecture with a fully connected layer designed to reduce the LSTM(128) down to a prediction of the five gestures used for analysis.

#### Data preprocessing

**Fig. 1:**
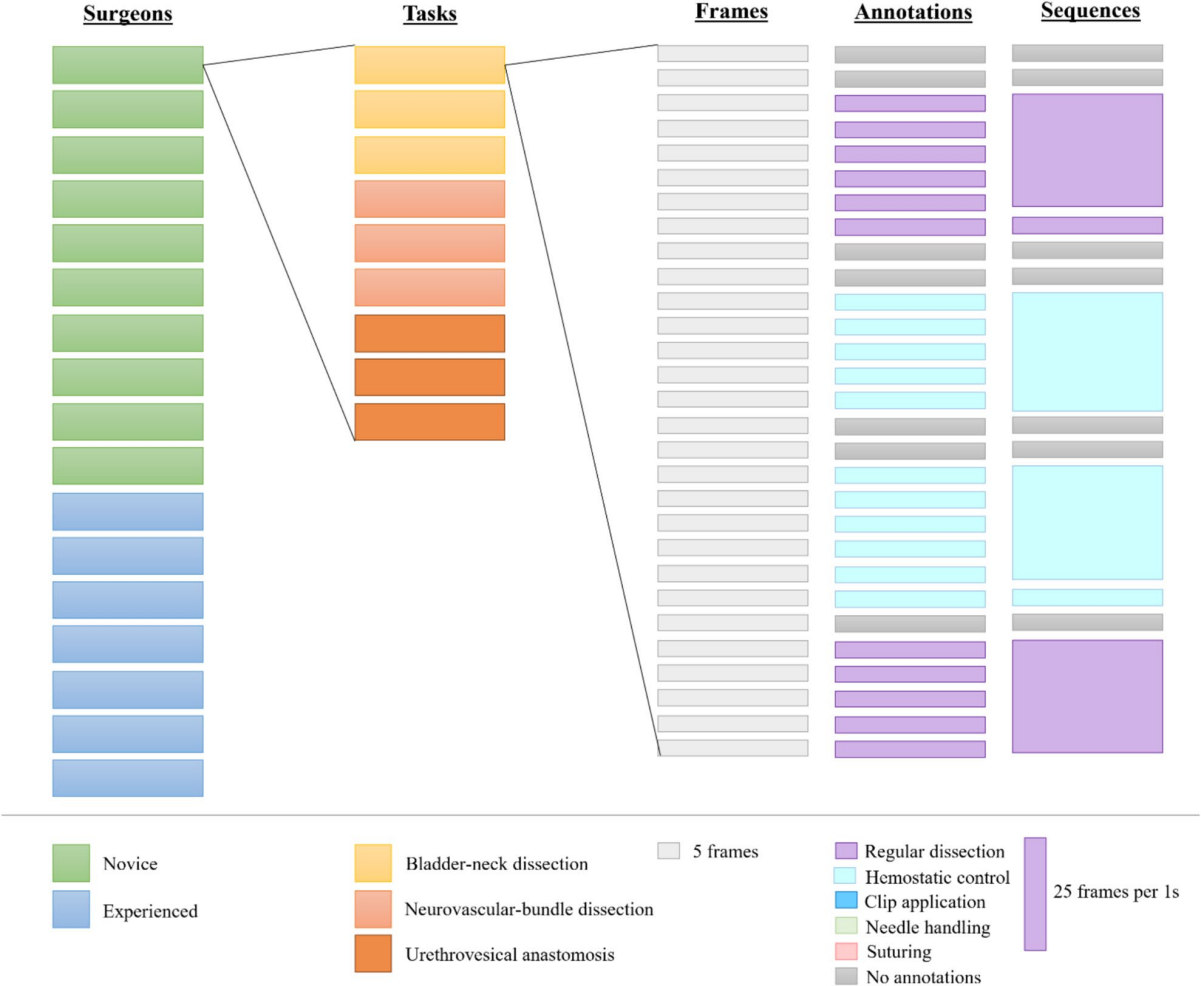
Pre-processing of the annotated videos from full videos into sequences of 25 frames per 1 s. The data set consisted of part procedures performed by novice or experienced RARP surgeons. Each surgeon performed each part of the procedure thrice. This part procedure was annotated and sampled at 25 frames per second

**Fig. 2:**
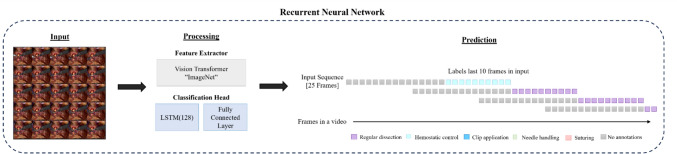
Overview of the model for automated surgical gesture annotation. Input: image sequences were pre-processed to 25 frames per second; Processing: the *feature extractor* reduced the number of dimensions in the frames while the *classification head* predicts the five gestures; Prediction based on the last 10 frames in a sequence, and each sequence is labeled with one of the five gestures. The feature extractor and classification header predict one gesture per input sequence*. LSTM: Long Short-Term Memory*

Before feeding the videos to the model, they were pre-processed into *input* sequences of 25 frames over 1 s (Fig. 1). All frames were normalized in color to match the requirements of the vision transformer and rescaled to a dimension of 299 × 299 pixels and three colors. These sequences were then used as input to the model (Fig. 2).

#### Model training

To train the network, the data set was split into a training + validation set and a test set with a sevenfold split to ensure the exclusion of video sequences from one experienced surgeon in each training and validation set, reserving the same ratio of experienced/novice videos for the test-set. This means that the dataset was divided into seven equal parts, wherein each fold, six parts were used for training and validation, and the remaining part was used for testing. This process was repeated seven times, ensuring that each subset served as the test set once.

The network was initially updated on the *training sets* and guided by the model's *validation set performance*. The *test set* was held out of the training processes and used to validate model performance. This ensures an unbiased prediction of the surgical gestures based on a dataset the network was not trained on and had never been presented with before.

#### Model inference

At inference, using the model for *prediction*, the actual usage of the trained model labeled every input sequence of all videos with one of the five surgical gestures (Fig. 2). During inference, we used a sliding window approach with a 15-frame overlap between sequences. For each input sequence (e.g., 25 frames), the model outputs predictions for all frames, but only the predictions for the last 10 frames in the sequence are retained. These are then appended to the overall prediction array. This approach ensures that each frame receives only one prediction, without the need for any additional post-processing to resolve overlaps.

**Fig. 3:**
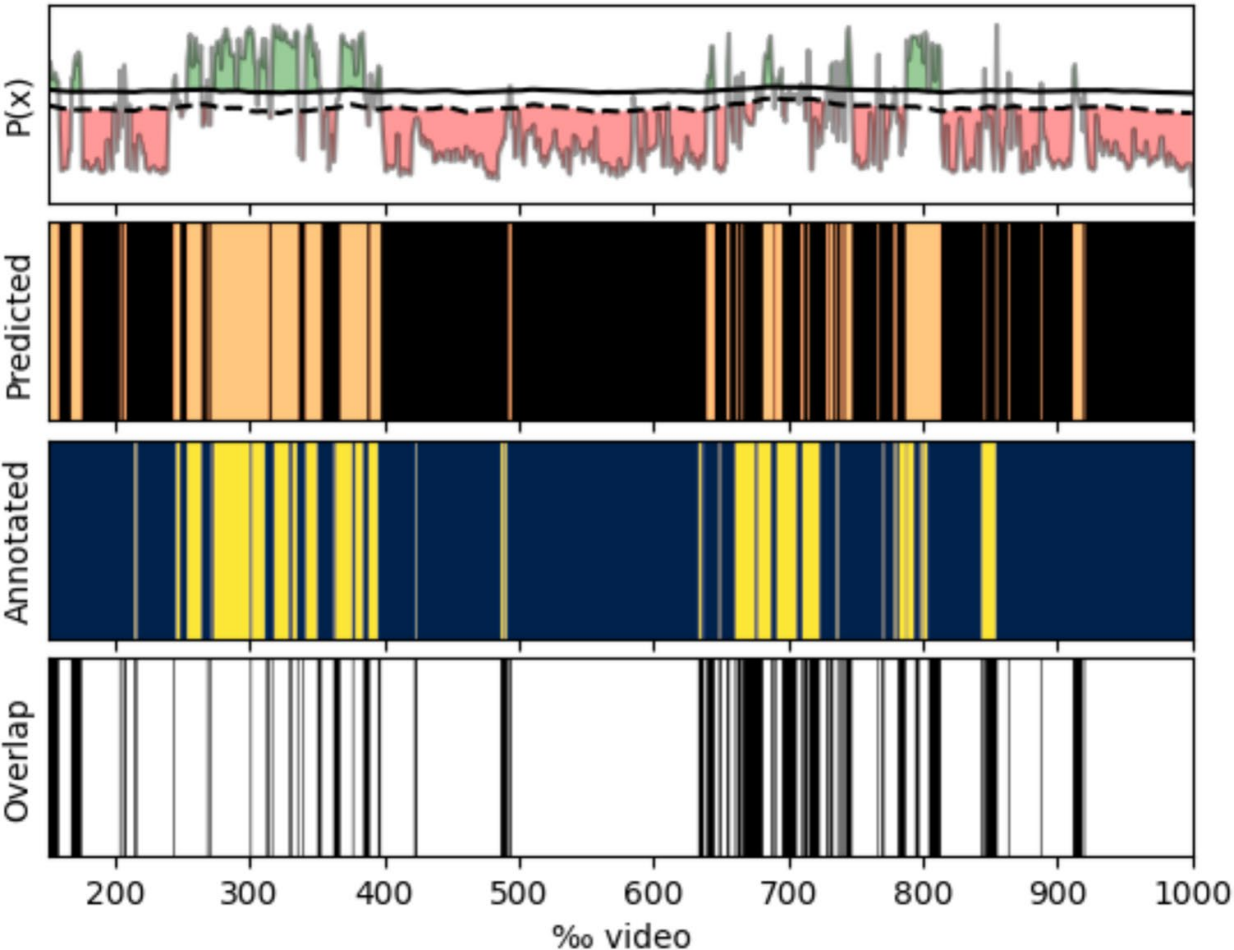
An example of the prediction of a gesture classification class for *Regular dissection* in *NVBD*. *The top panel* shows the model's probability output, P(x), over time. The solid black line represents the smoothed probability signal, while the dashed black line indicates the decision threshold used to classify gestures. Areas shaded green correspond to segments where the probability exceeds the threshold (i.e., predicted presence of the gesture), and areas shaded red indicate segments below the threshold (i.e., absence of the gesture). *The second panel* displays the Predicted gesture labels over time, where orange segments represent predicted gesture occurrences, and black segments indicate no gesture predicted. *The third panel* shows the Annotated ground truth labels, with yellow segments representing annotated gesture occurrences and dark blue segments indicating no gesture. *The bottom panel* visualizes the Overlap, with black bars marking frames where the prediction matches the ground truth and white areas indicating mismatches. The x-axis reflects normalized video progress in per mille units (‰ video).

Using Softmax activation, the model predicted the probability of each of the five gestures being present in a sequence (Fig. 3). When all predictions were made, the probability of a gesture being present was updated according to Bayes’ theorem. The gesture with the highest probability in the sequence was labeled, so only one gesture was labeled for each input sequence.

#### Post-processing: from sequences to segmented video

The network's input was the whole video, normalized to a fixed length of 1000 to easily handle videos of varying lengths consistently. The analysis employs an adaptive thresholding approach based on entropy to enhance prediction precision and dynamic event detection (Fig. 3). The entropy-based thresholding operates by calculating a sliding window entropy over the predictions from the model, adjusting the threshold adaptively to account for local variations in prediction uncertainty. Higher entropy results in a more relaxed threshold, while lower entropy enforces stricter criteria. Hysteresis thresholding was applied using dynamic high and low thresholds derived from entropy-adjusted thresholds. The high threshold is set slightly above the entropy-based threshold (+ 0.1), while the low threshold is set slightly below (− 0.1). This two-level thresholding enables robust event detection by accounting for transient, high-frequency fluctuations in predictions.

#### Visual comparison of correct and incorrect predictions

To qualitatively assess model performance, we generated visual comparisons between misclassified and correctly classified sequences. For each misclassified example, a correctly classified sequence of the same class label and from the same video was identified. Matching was performed by comparing filename-encoded metadata: class label and video ID were required to be identical, and the sequence number was allowed to vary within a range of ± 10 sequences (250frames, ~ 10 secs) to find a close temporal match. Only unused correct examples were considered to avoid repetition.

Each video sequence was represented as a fixed-size image palette composed of 25 consecutive RGB frames arranged in a grid. These palettes were saved both as standalone images and embedded in a multi-page PDF to support side-by-side visual inspection (Supplementary File).

### Statistical analysis

The overall performance of the neural network was assessed by multiple commonly used metrics for multi-label classification: Area Under the Curve (AUC) for assessing true positive rate against false positive rate and F1-score for determining the harmonic mean of precision (Positive predictive value) and sensitivity (True positive rate).

Performance for each of the five surgical gesture classifications was assessed using metrics for single-label classification: Sensitivity (True positive rate), Specificity (True Negative rate), and Accuracy (overall proportion of correct predictions, both true positives and true negatives).

Performance on the video level: The Intersection over Union (IoU) is a common metric used to evaluate the similarity between two arrays, typically in the context of temporal segmentation tasks. In the standard definition, the IoU is calculated as the ratio of the intersection (overlapping positive/true elements) to the union (total unique positive elements) of the two arrays. However, this conventional approach ignores cases where both prediction and annotations contain zero values – a situation that is also desirable. To account for this, we defined *Total Agreement*, an extended version of IoU, which incorporates both matching positive values and zero matching into the intersection and union calculations.

The models and statistical analysis were performed using the Python programming language (version 3.10.10, Python Software Foundation, Amsterdam, Netherlands, https://www.python.org/ and the libraries OpenCV, Numpy, Scipy, Tensorflow, Keras-vit, and Matplotlib.

## Results

We used 161 videos of three partial procedures (*Bladder-neck dissection*: 55 videos, *Neurovascular bundle dissection*: 54 videos, *Urethrovesical anastomosis*: 52 videos), totaling 2565 min in length. We annotated 6,550 gestures across the videos with an average gesture length of 9.6 s. In the data set, *Regular dissection* was the most common gesture annotated (2,461 annotations), followed by *Needle handling* (1,517 annotations), *Hemostatic control* (1,229 annotations), *Suturing* (1,035 annotations), and *Applications of clips* (308 annotations).

**Fig. 4:**
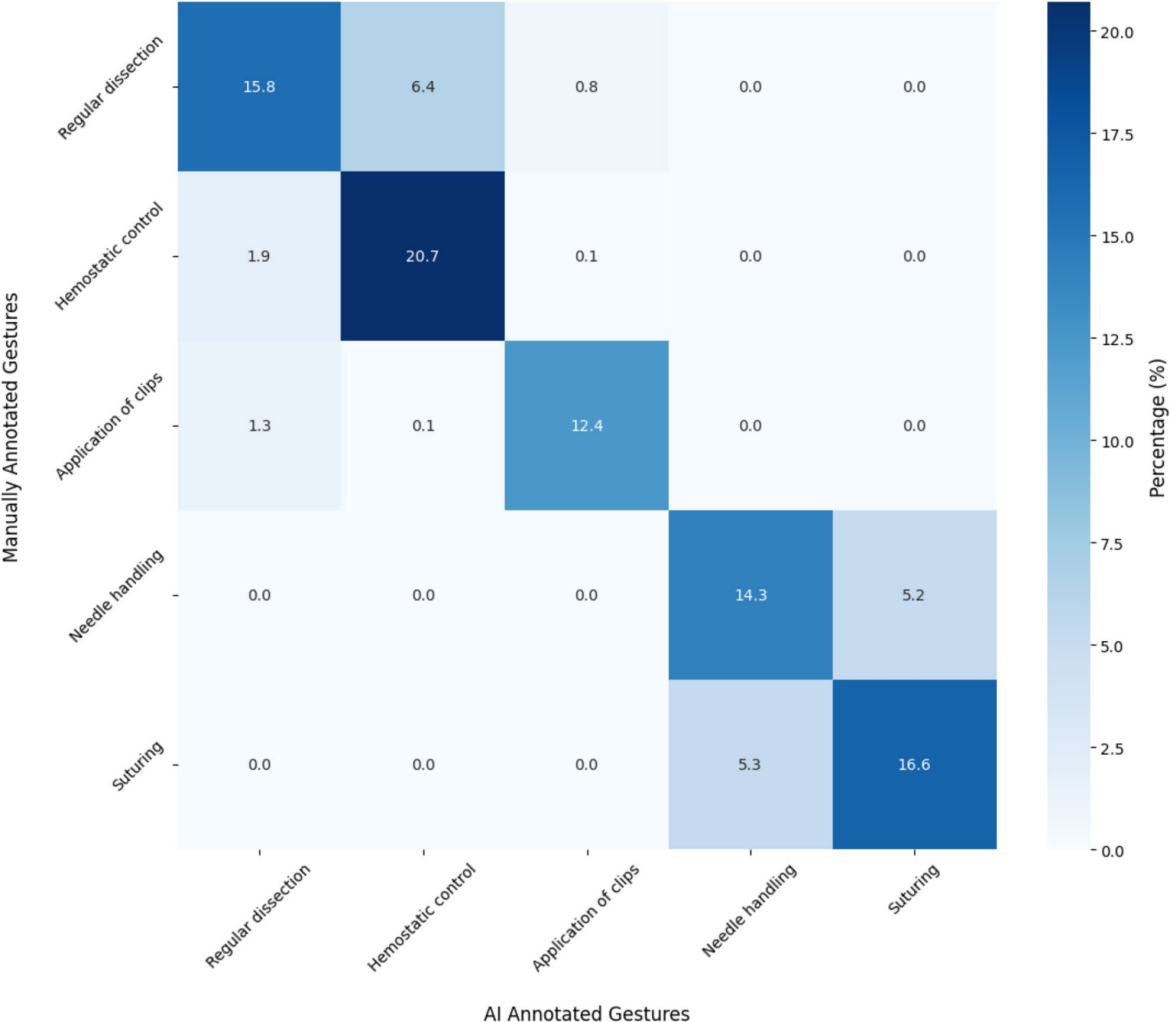
Confusion Matrix showing the prediction of each class of surgical gesture with a total distinction between *dissection* gestures (Regular dissection, Hemostatic control, and Clip application) and *suturing* gestures (Needle handling and Suturing). Numbers are percentages, calculated on the test-set results from the K-fold cross validation (k = 7). Folds contained sample number of 1051.2 (1009–1078) [average (min—max)]

In a multi-labeling analysis, our neural network was able to predict the class of surgical gestures with an Area Under the Curve (AUC) of 0.95 (95% CI 0.93–0.96) and an F1-score of 0.71 (95% CI 0.67–0.75). The network showed strong performance in classifying each surgical gesture with accuracies > 0.8 (0.84–0.97) and high specificities (0.90–0.99) but with lower sensitivities (0.62–0.81). The network classified all the *dissection gestures* with high specificity, sensitivity, and accuracy. The model found it more challenging to distinguish the two suturing gestures accurately as the specificity, sensitivity, and accuracy were lower than the *dissection gestures* (Table 1). However, the network has learned to fully distinguish *dissection gestures* from *suturing gestures* with no overlap in classification between the two gesture groups (Fig. 4).

### Video segmentation into gestures

The Total Agreement metric for segmenting videos into surgical gestures revealed patterns when stratified by part-procedure (BND, NVBD, or UVA) and gesture class. When stratified by procedure, the average Total Agreement was 0.81 (95% CI ± 0.03) for BND, 0.84 (95% CI ± 0.02) for NVBD, and 0.84 (95% CI ± 0.03) for UVA.

We stratified the data by gesture class. The average Total Agreement for *Hemostatic control* for the dissection gesture class was 0.77 (95% CI ± 0.04). *Regular dissection* had an average Total Agreement of 0.72 (95% CI ± 0.03), while the *Application of clips* showed a higher average Total Agreement of 0.91 (95% CI ± 0.02). For the *suturing* gesture class, the *Needle handling* class showed an average Total Agreement of 0.86 (95% CI ± 0.03), and *Suturing* achieved 0.88 (95% CI ± 0.03).

We combined part-procedure type and gesture class. For procedure-part BND, *Hemostatic control* had a Total Agreement average of 0.43 (95% CI ± 0.03), *Regular dissection* averaged 0.63 (95% CI ± 0.03), and *Application of clips* had a high average of 0.99 (95% CI ± 0.00). For part-procedure NVBD, the *Hemostatic control* displayed an average Total Agreement of 0.90 (95% CI ± 0.03), *Regular dissection* averaged 0.57 (95% CI ± 0.03), and *Application of clips* had an average of 0.74 (95% CI ± 0.03). No *suturing* gestures were performed in BND and NVBD causing *Needle handling* and *Suturing* to show near-perfect agreement with averages of 1.00 (95% CI ± 0.00). Within part-procedure UVA, as expected, *Hemostatic control, Regular dissection,* and*, Clip application* had an average Total Agreement of 1.00 (95% CI ± 0.00) as *dissection* gestures are not performed in this part-procedure. The *Needle handling* and *Suturing* gesture classes exhibited averages of 0.56 (95% CI ± 0.02) and 0.62 (95% CI ± 0.02).

## Discussion

Accurate prediction of patient outcomes in the clinical setting using AI models relies on robust models trained on large, diverse datasets. This is not feasible to implement within a reasonable timeframe if the surgical gesture annotation used for surgical analysis must be performed manually. The first step in solving this is to automate the surgical gesture analysis, as the manual annotation process is very time-consuming and resource-demanding [16]. To address this challenge, we have created a high-performing model for automated surgical gesture identification, achieving a class prediction accuracy with an AUC of 0.95. The model can identify five distinct gestures in dissection and suturing across three partial procedures of the RARP with high accuracies of > 0.8 and specificities of ≥ 0.9. Further, as a novel feature, the network was designed to timestamp each identified gesture, marking the beginning and end of each surgical gesture within the surgical video. By automating this process, the model eliminates the need for manual labeling, which traditionally requires substantial time and experience to annotate the correct timestamp for each surgical gesture precisely. This advancement ensures that gesture data is accurately mapped to the surgical timeline, enabling a more detailed analysis of the surgical workflow, efficiency, and use of the different surgical gestures.

The performance of our model is high; however, we chose to train the model on simulation-based videos of RARP surgery instead of real-life procedures.

Simulation-based training has the advantage of a standardized environment with no anatomy or case complexity change [17, 18]. This makes it possible to perform gesture analysis on surgeons at all experience levels, from novices to highly experienced RARP surgeons. As we have previously shown, surgeons express different patterns of surgery, utilizing specific gestures in multiple ways, which might strengthen the neural network's accuracy when presented with various work patterns [10]. Furthermore, it is easier to access simulation-based surgeries, as they only require consent and time-use from the surgeon and are not dependent on the operating room schedule and patient availability. An experienced surgeon can perform the number of surgeries thrice in a simulation-based environment compared to the real world, enabling access to more data in a shorter amount of time, which will benefit the performance of the models.

We acknowledge that even though the model performs well in this standardized setting, it still needs to be tested on real-life surgeries. Our next step is to transfer the model to gesture analysis on real-life RARP. Our theory is that we can use the model we have now created as a base for training automated gesture analysis on real-life surgeries. We hope it will require fewer real-life surgeries to accomplish a good performance of the model, as the model has already been presented with gesture analysis in a similar environment. Gonzales et al. [19] have shown that machine learning models on basic simulator tasks can be applied to real-life surgeries with an increase in classification accuracy from 93 to 95.4% and Cui et al. [8] found that gesture patterns in basic simulated tasks could predict patient outcomes in RARP. Simulated tasks may contain enough information to train an initial neural network and then transfer it to real-life surgeries with minor adjustments. In contrast, Itzkovich et al. [20] found that LSTM networks trained on basic simulated tasks could not be used in real-life surgeries. Basic simulated tasks do not have the advantages of procedure-specific simulation-based tasks, which are similar in anatomy and surgical knowledge. Therefore, our hope is that our model can be used for real-life surgeries. However, to our knowledge, this has not been tested before for surgical gesture analysis.

Using simulated videos is just one way to reduce the need for large datasets. Another way could be to use available data sets online. However, most available datasets only cover a few different procedures, and most data sets only have simulated basic tasks. If we all create our algorithms on these data sets, we will lack the diversity that inevitably exists across surgical procedures, institutions, and surgeon experience levels. With the lack of big datasets and the acquisition of these, the entire field is still stalled by the lack of data sets available for building robust models [11]. This field of research is still novel.

A few studies have connected surgical gesture analysis with patient outcomes in RARP surgery, where gesture analysis can predict both erectile dysfunction and incontinence [6–8, 21]. However, multiple areas still need to be investigated for this field to move forward: the optimal use and acquisition of data for surgical gesture analysis and model performance. We want our models to predict as close to the ground truth as possible, but the models depend on the data it is being trained on. Even with artificial intelligence algorithms' opportunity to bypass some of the clinical inter-surgeon bias, the data will still be biased from the surgeries we choose to feed the model to the skill of the annotator. Some of these issues can be solved by feeding high-quality data to our models. However, we still do not know how well these models must perform to outperform the surgeons' predicted outcomes and be a valid tool for decision-making for both surgeons and patients.

Our research relied on data from a single institution using simulation-based part-procedures instead of real-life full-length procedures. Further, only one annotator performed the annotations, which could reduce the generalizability of the data set. Only part-procedures of RARP were available on the simulator at the time of the study. The partial procedures we selected represent some of the most technically challenging and critical phases of RARP, making them highly relevant for evaluating model performance. Additionally, annotating full-length procedures, which often exceed three hours, is extremely time-consuming and remains largely unexplored in the literature for this reason. While we acknowledge that full-procedure analysis may offer broader insights, the field currently lacks evidence on whether this is necessary or advantageous compared to focusing on key segments. Although our model performed well, we still need to evaluate its effectiveness in real-life surgeries and its ability to predict outcomes. This is just the initial step toward automating surgical gesture annotation and advancing the field.

In the era of 'Big Data,' we face an overwhelming amount of information, so making effective patient decisions is crucial. Treatment decisions depend on various factors, including examinations, tests, scans, and patient preferences. While tools that integrate relevant knowledge are needed, they are not yet fully incorporated into clinical practice and require validation across multiple institutions. Surgeons must also understand the benefits and limitations of these tools to make informed decisions with their patients.

## Conclusion

We trained a high-performing neural network for automated gesture annotation using simulation-based robot-assisted radical prostatectomies. The network performed well for both video sequences and full-length videos. The next step is to transfer the network to real-life surgeries and test the automated annotations’ ability to predict patient outcomes.

## Supplementary Information

Below is the link to the electronic supplementary material.

Supplementary file1 (PDF 9969 KB)

## Data Availability

No datasets were generated or analysed during the current study.
