# Supplementary material for: Development of an artificial intelligence algorithm for automated surgical gestures annotation
Source: J Robot Surg. 2025 Jul 18;19(1):404. doi: 10.1007/s11701-025-02556-2 (PMC12274238; doi:10.1007/s11701-025-02556-2)

True Class Regular Dissection

True Positive: Regular Dissection
rarpexp001B1_93_C0_X1.npy
(data)

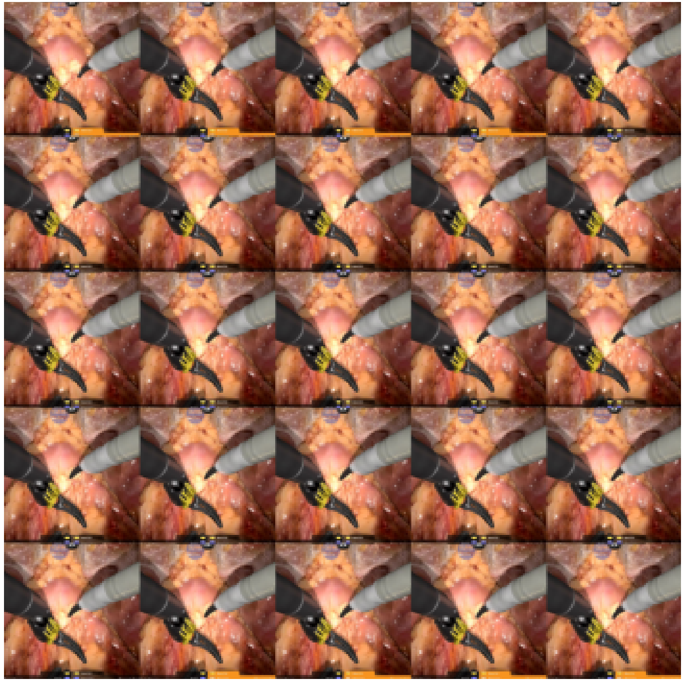

False Negative: Regular Dissection
rarpexp001B1_103_C0_X1.npy
(data)

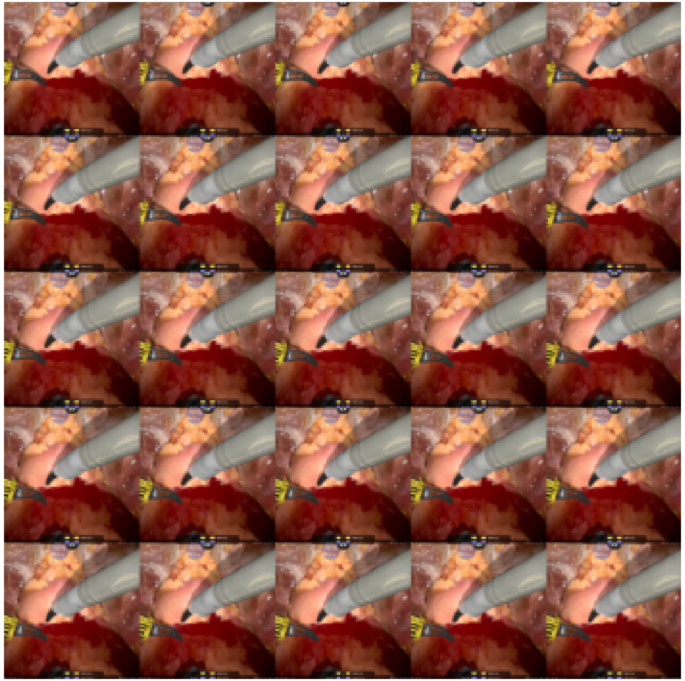

True Class Regular Dissection

True Positive: Regular Dissection
rarpexp001B1_108_C0_X1.npy
(data)

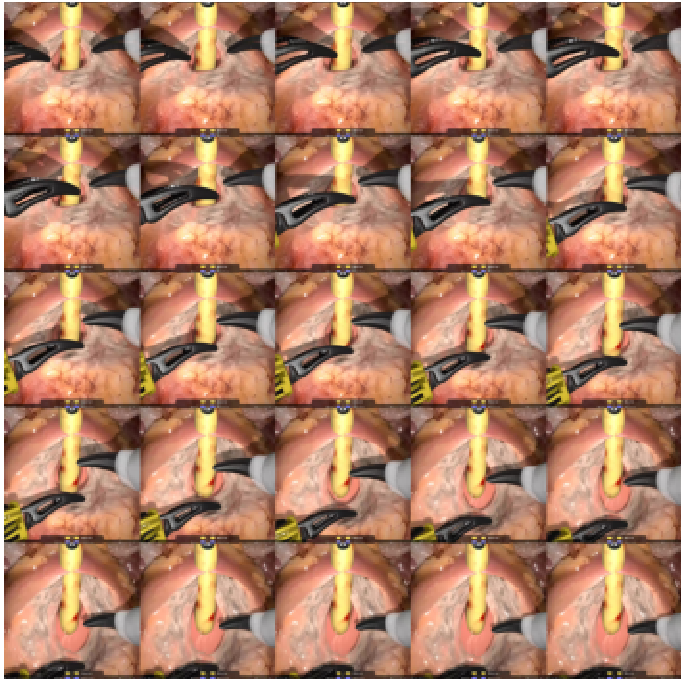

False Negative: Regular Dissection
rarpexp001B1_118_C0_X1.npy
(data)

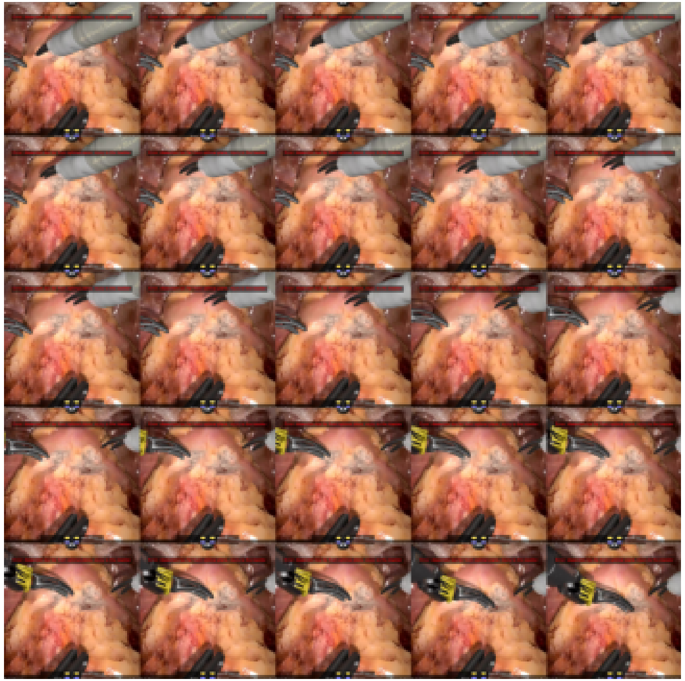

True Class Regular Dissection

True Positive: Regular Dissection
rarpexp002B2_9_C0_X1.npy
(data)

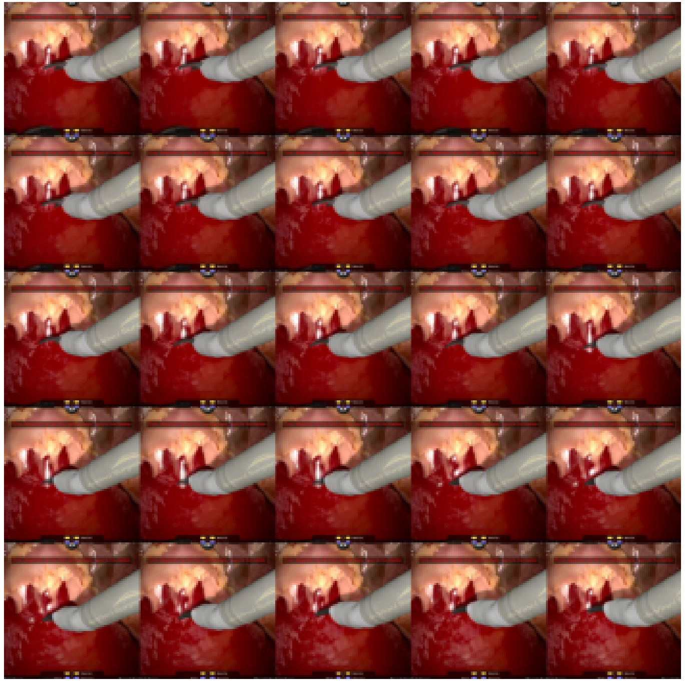

False Negative: Regular Dissection
rarpexp002B2_17_C0_X1.npy
(data)

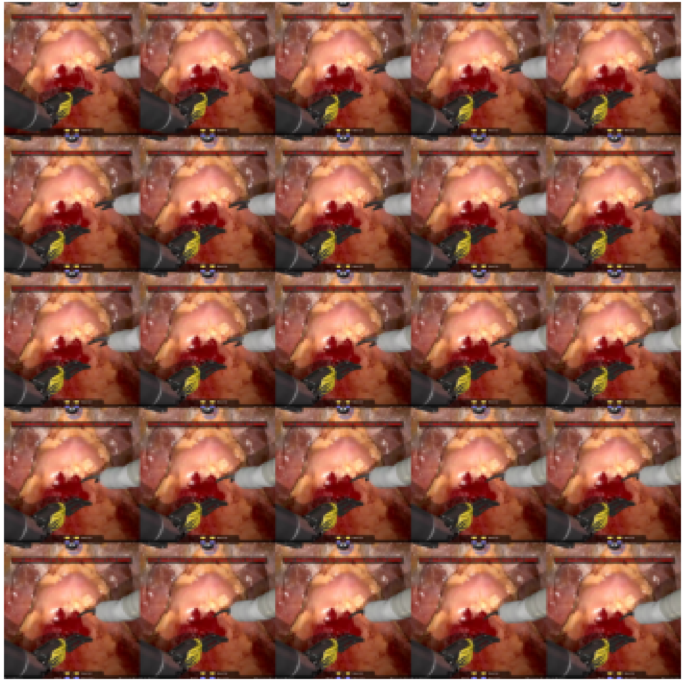

True Class Regular Dissection

True Positive: Regular Dissection
rarpexp002B2_187_C0_X1.npy
(data)

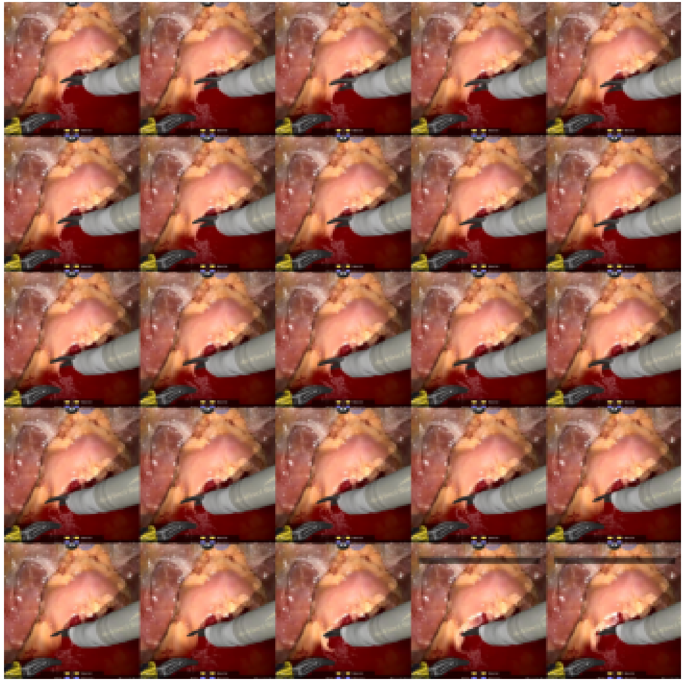

False Negative: Regular Dissection
rarpexp002B2_188_C0_X1.npy
(data)

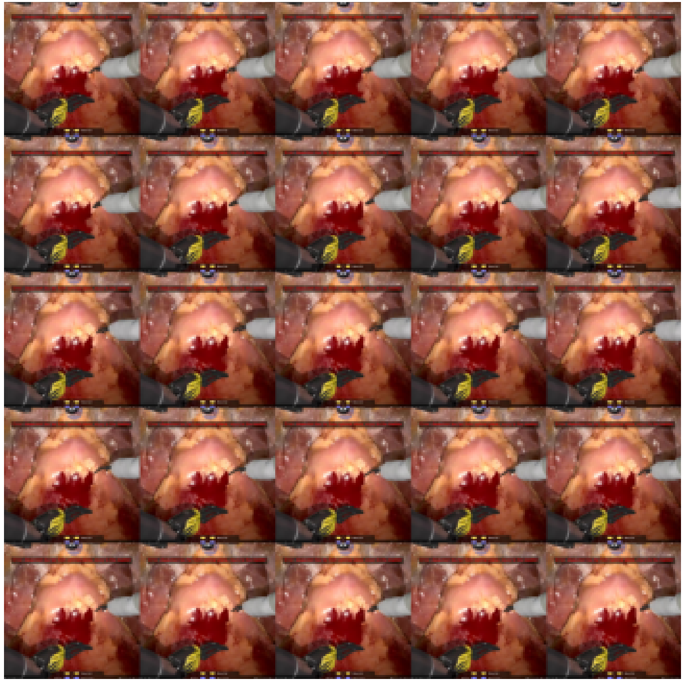

True Class Haemostasis

True Positive: Haemostasis
rarpexp002B1_9_C0_X0.npy
(data)

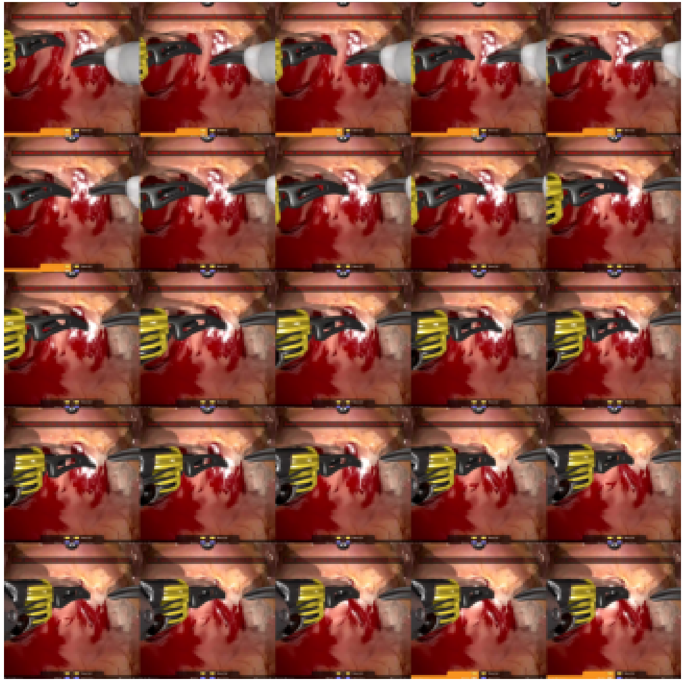

False Negative: Haemostasis
rarpexp002B1_5_C0_X0.npy
(data)

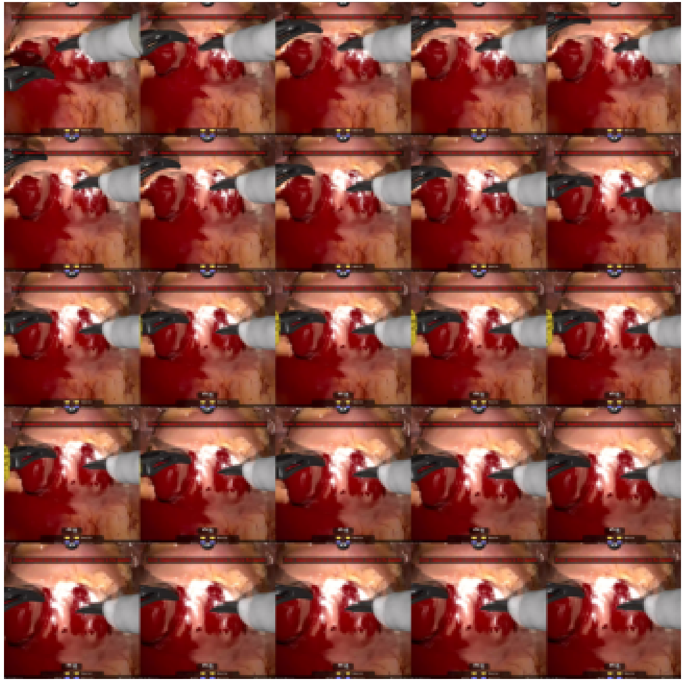

True Class Regular Dissection

True Positive: Regular Dissection
rarpexp002B1_68_C0_X1.npy
(data)

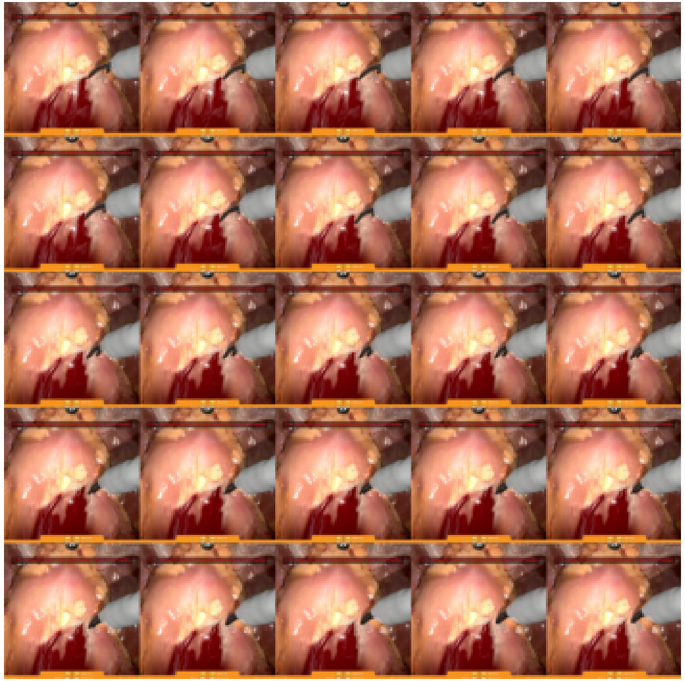

False Negative: Regular Dissection
rarpexp002B1_69_C0_X1.npy
(data)

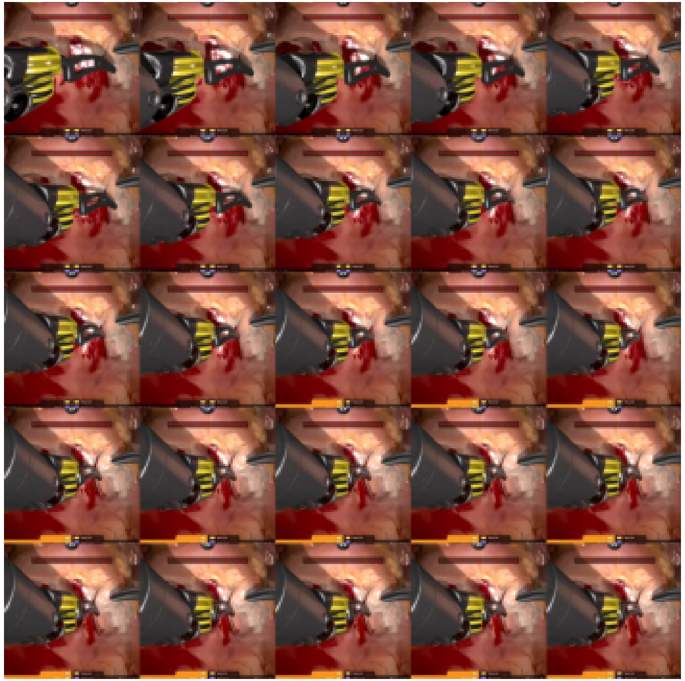

True Class Haemostasis

True Positive: Haemostasis
rarpexp002B1_11_C0_X0.npy
(data)

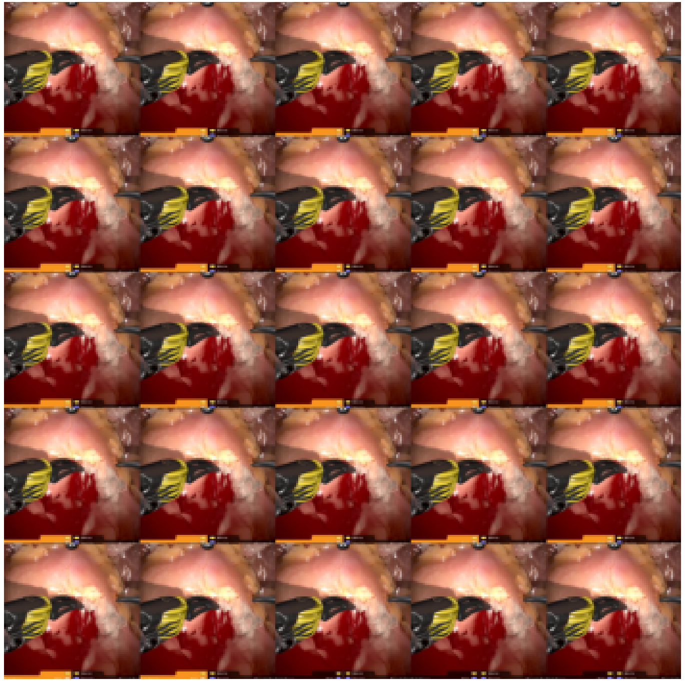

False Negative: Haemostasis
rarpexp002B1_6_C0_X0.npy
(data)

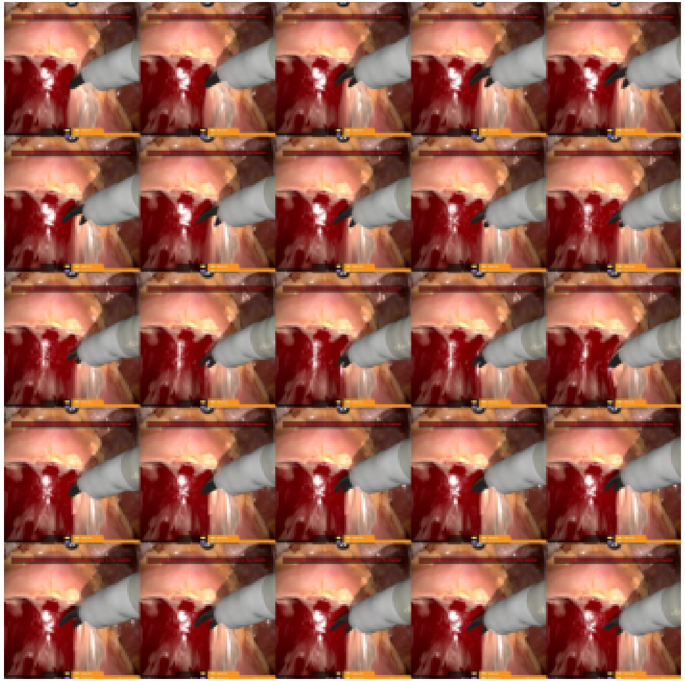

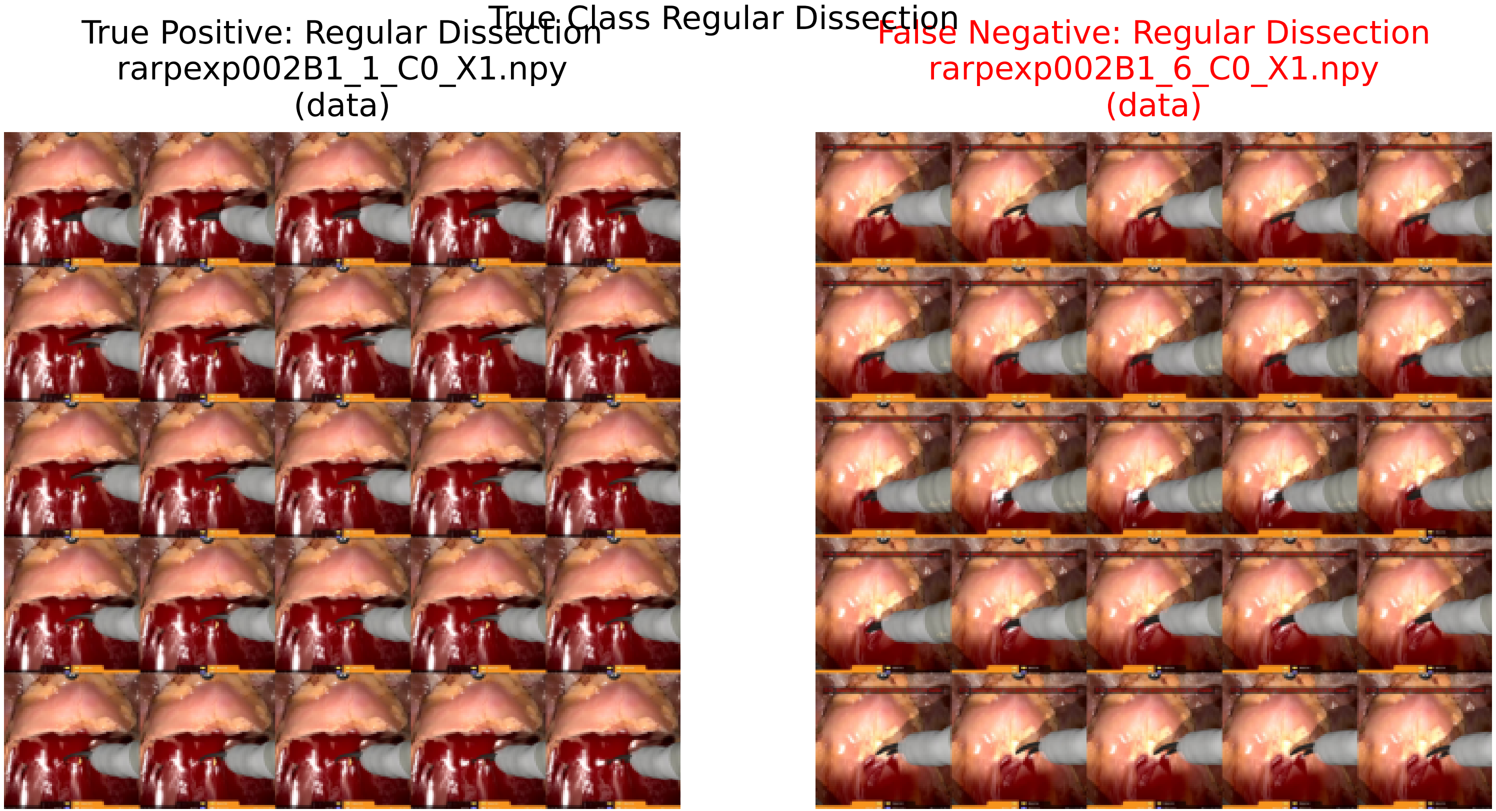
True Class Regular Dissection
True Positive: Regular Dissection
rarpexp002B1_1_C0_X1.npy
(data)
False Negative: Regular Dissection
rarpexp002B1_6_C0_X1.npy
(data)

True Class Regular Dissection

True Positive: Regular Dissection
rarpexp002B1_74_C0_X1.npy
(data)

False Negative: Regular Dissection
rarpexp002B1_70_C0_X1.npy
(data)

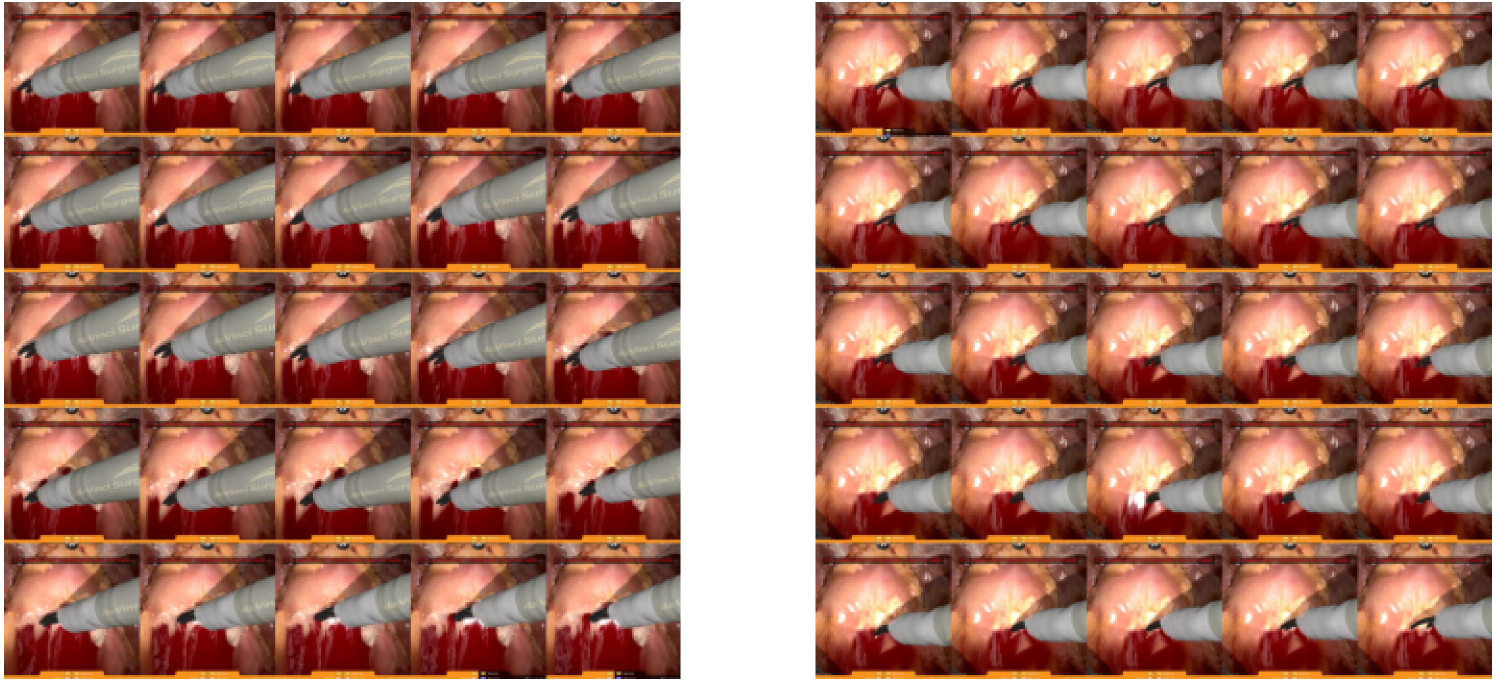

True Class Regular Dissection

True Positive: Regular Dissection
rarpexp002B2_46_C0_X1.npy
(data)

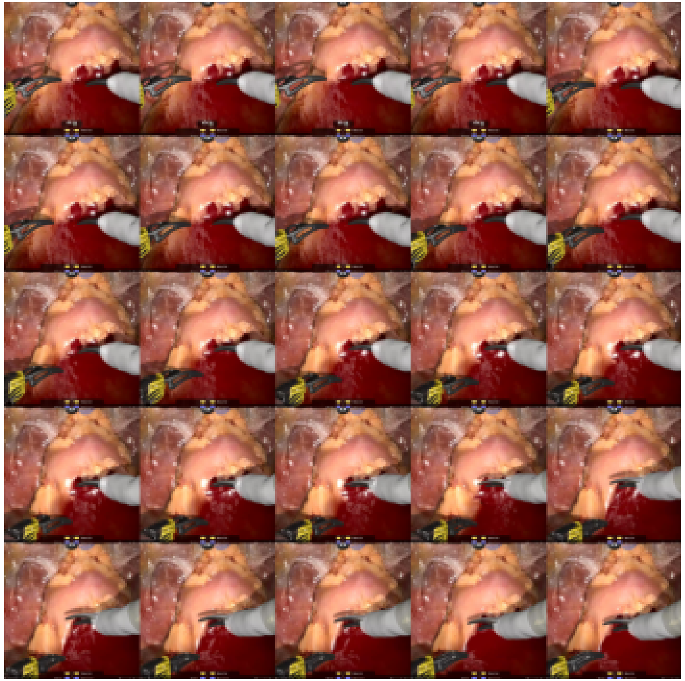

False Negative: Regular Dissection
rarpexp002B2_54_C0_X1.npy
(data)

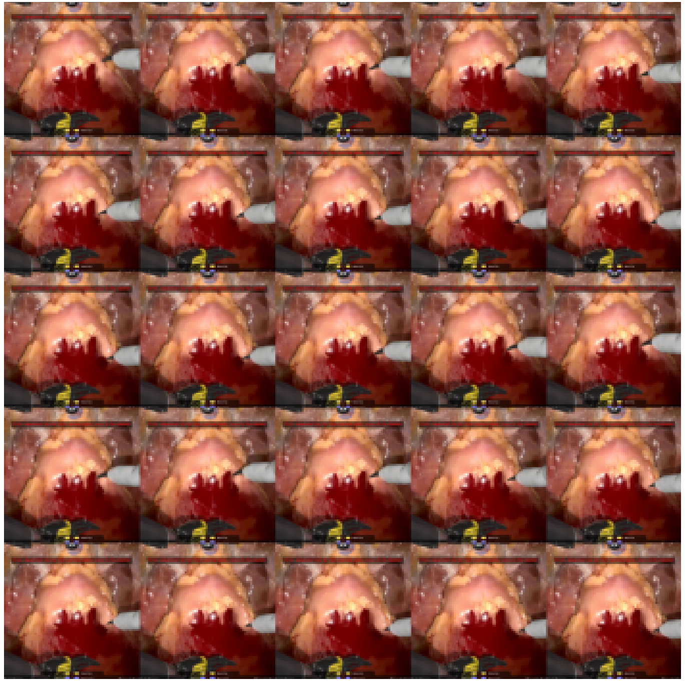

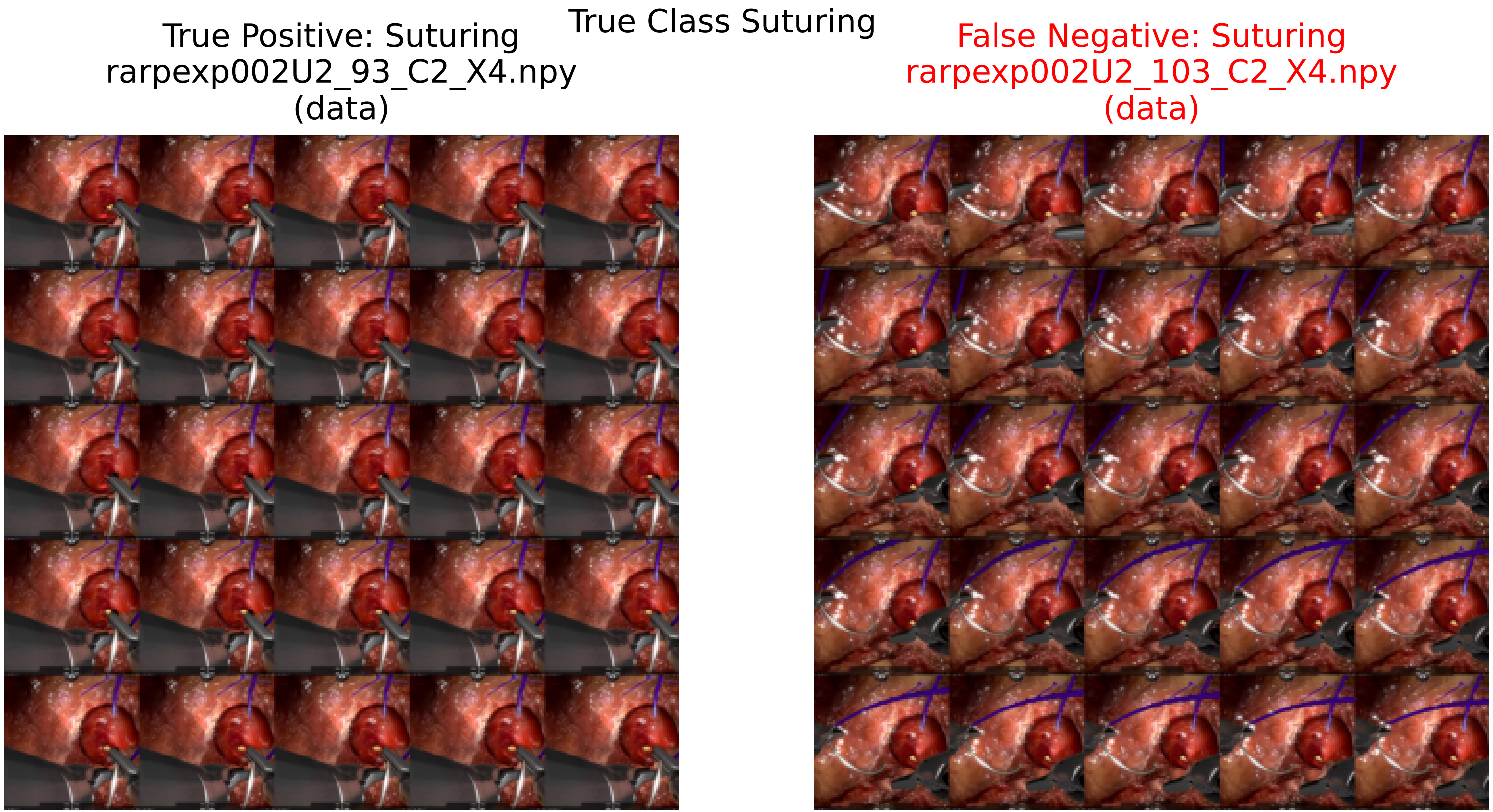
True Class Suturing
True Positive: Suturing
rarpexp002U2_93_C2_X4.npy
(data)
False Negative: Suturing
rarpexp002U2_103_C2_X4.npy
(data)

True Class Suturing

True Positive: Suturing
rarpexp002U2_145_C2_X4.npy
(data)

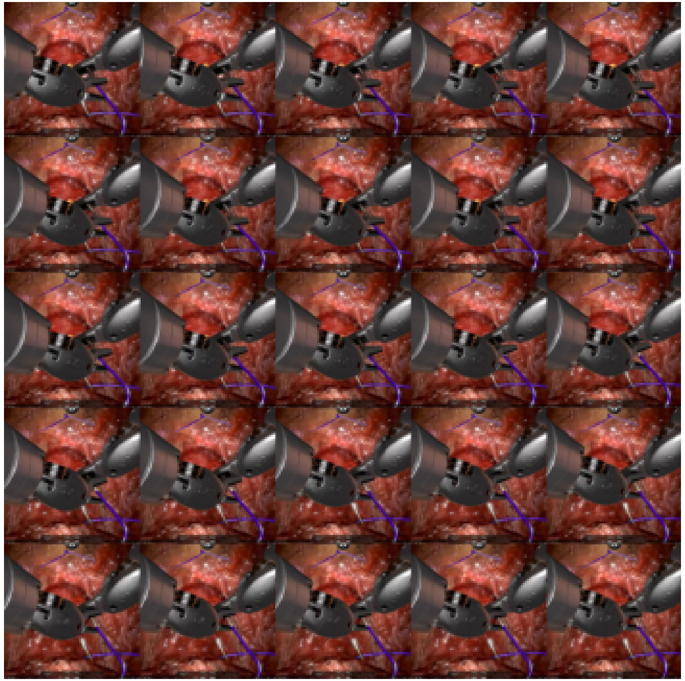

False Negative: Suturing
rarpexp002U2_155_C2_X4.npy
(data)

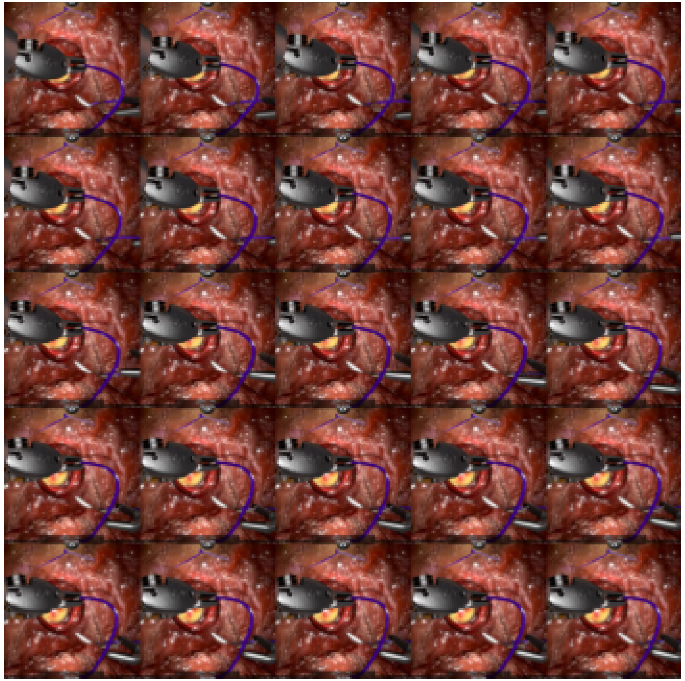

True Class Suturing

True Positive: Suturing
rarpexp004U1_92_C2_X4.npy
(data)

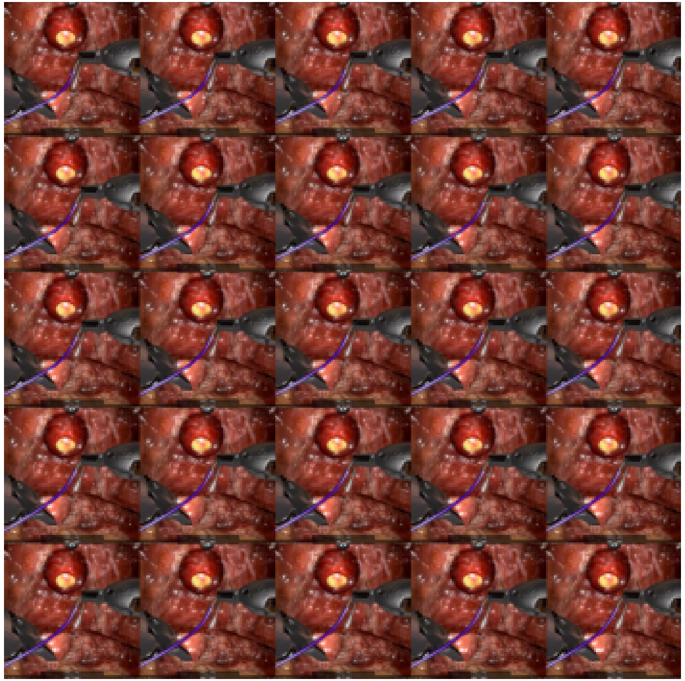

False Negative: Suturing
rarpexp004U1_101_C2_X4.npy
(data)

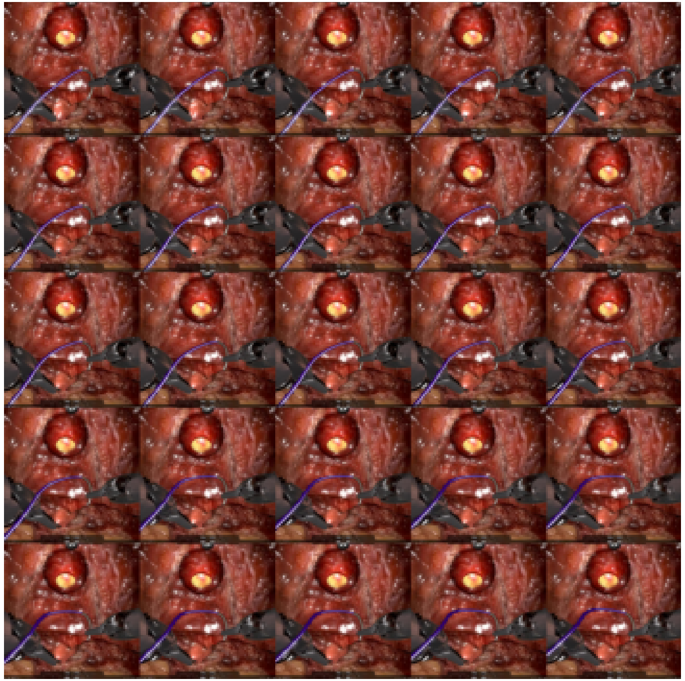

True Class Suturing

True Positive: Suturing
rarpexp004U1_9_C2_X4.npy
(data)

False Negative: Suturing
rarpexp004U1_10_C2_X4.npy
(data)

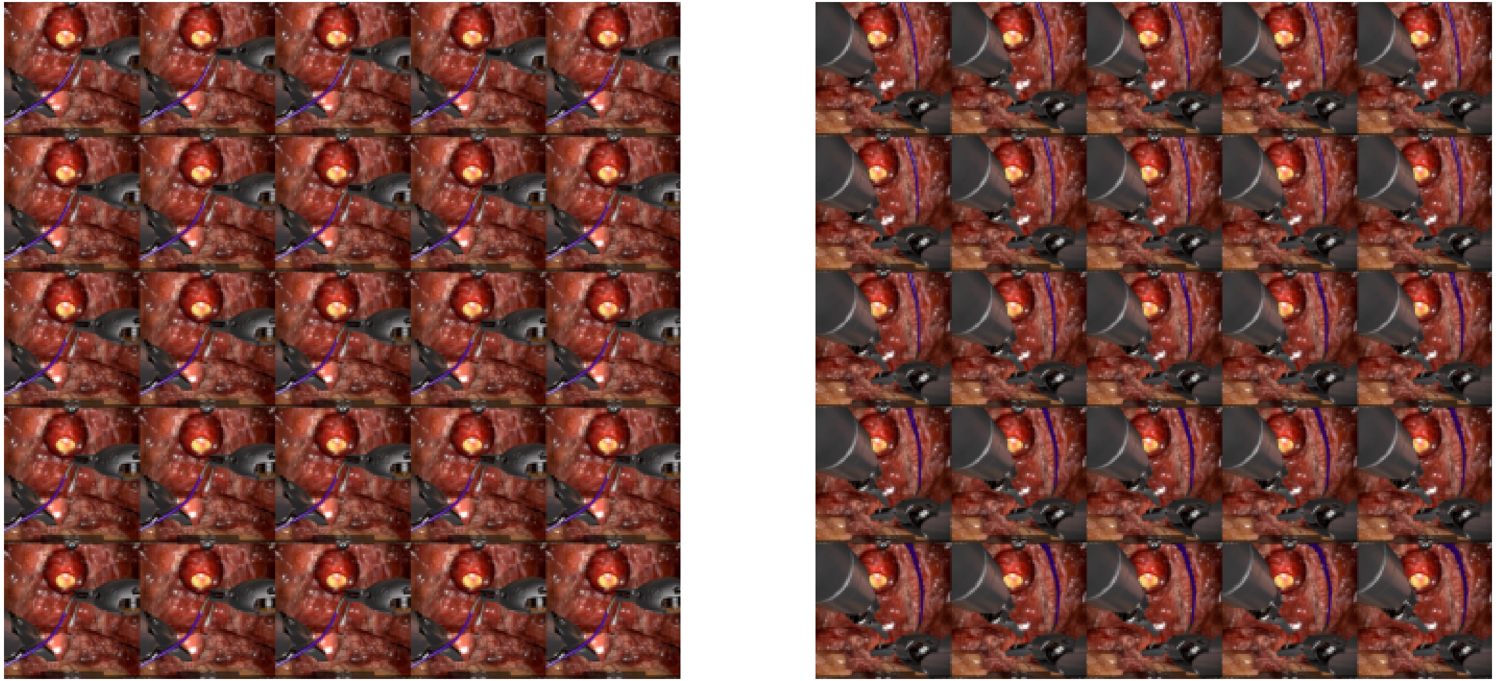

True Class Suturing

True Positive: Suturing
rarpexp004U1_108_C2_X4.npy
(data)

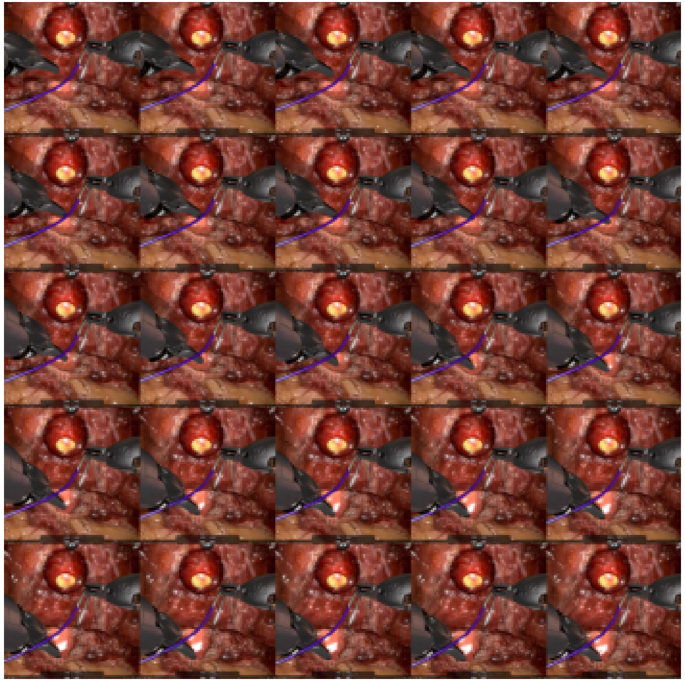

False Negative: Suturing
rarpexp004U1_112_C2_X4.npy
(data)

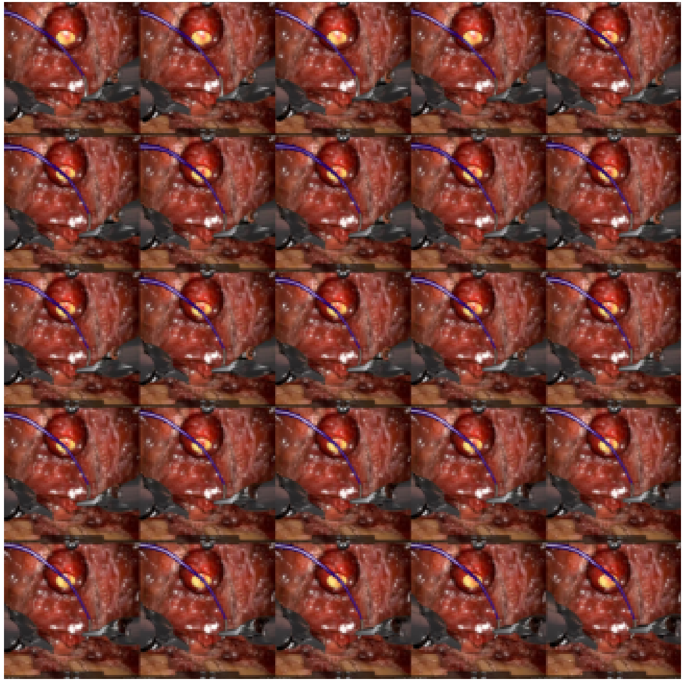

True Class Suturing

True Positive: Suturing
rarpexp004U1_123_C2_X4.npy
(data)

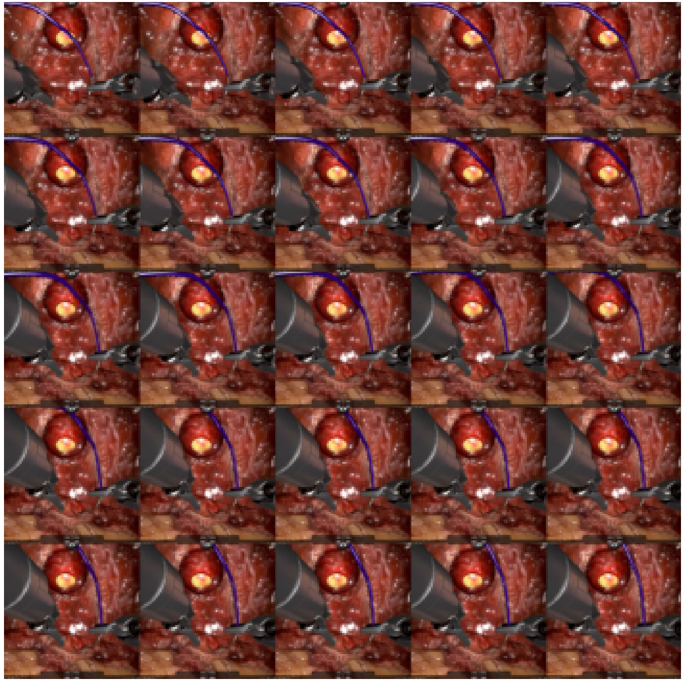

False Negative: Suturing
rarpexp004U1_113_C2_X4.npy
(data)

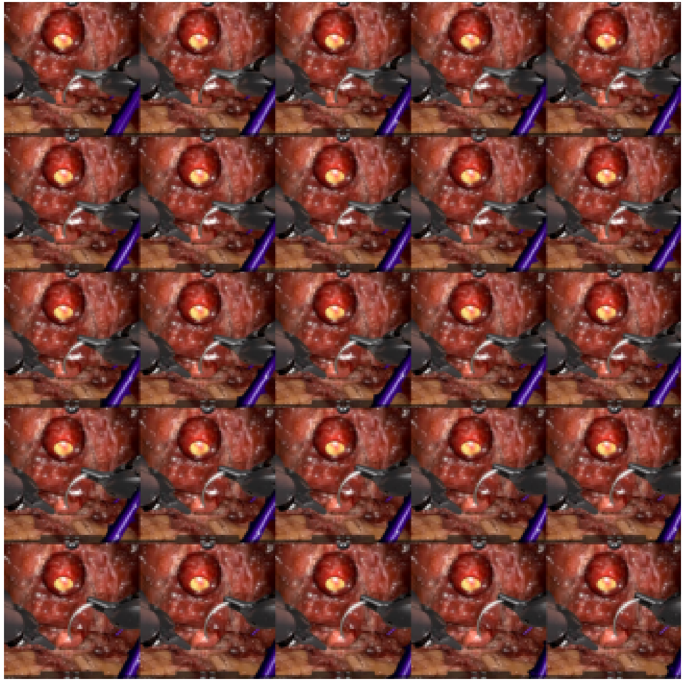

True Class Suturing

True Positive: Suturing
rarpexp002U2_15_C2_X4.npy
(data)

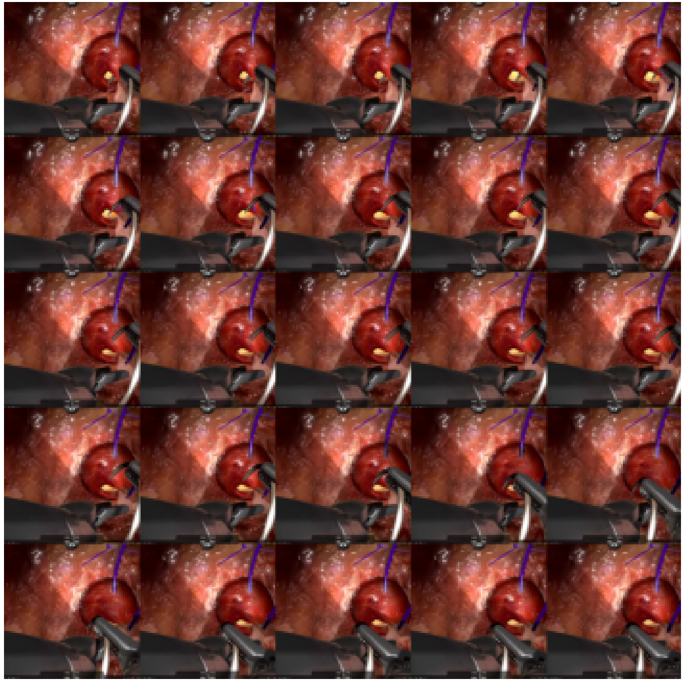

False Negative: Suturing
rarpexp002U2_25_C2_X4.npy
(data)

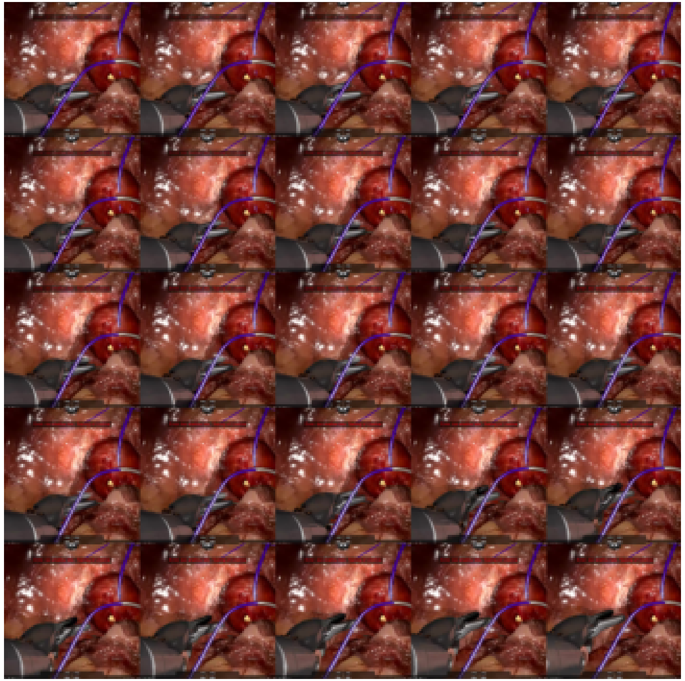

True Class Suturing

True Positive: Suturing
rarpexp002U2_20_C2_X4.npy
(data)

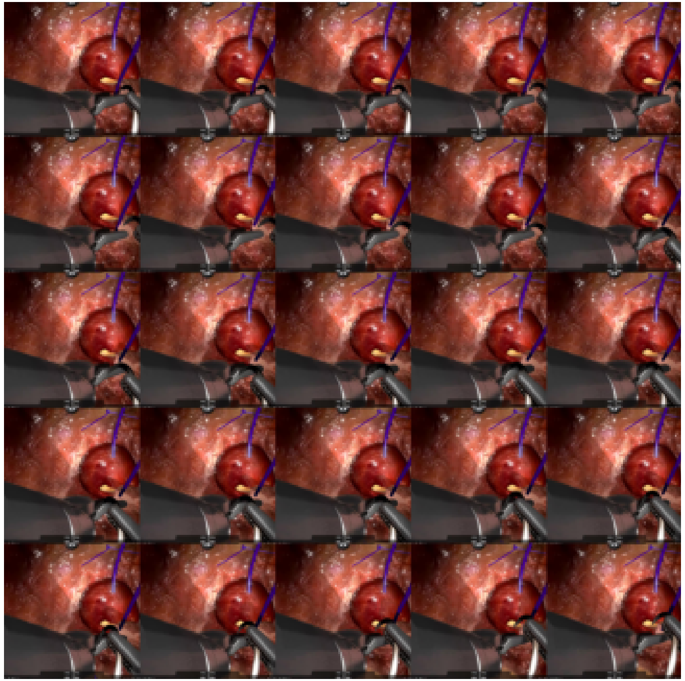

False Negative: Suturing
rarpexp002U2_27_C2_X4.npy
(data)

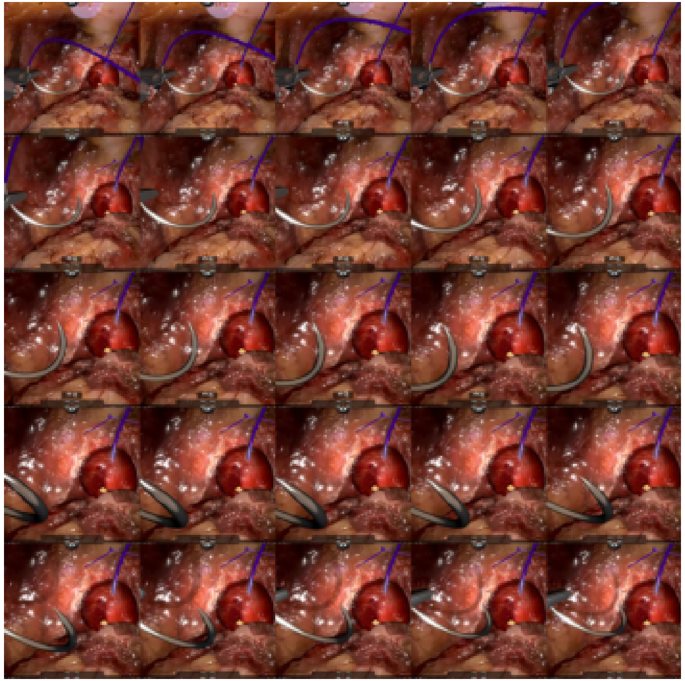

True Class Suturing

True Positive: Suturing
rarpexp002U2_21_C2_X4.npy
(data)

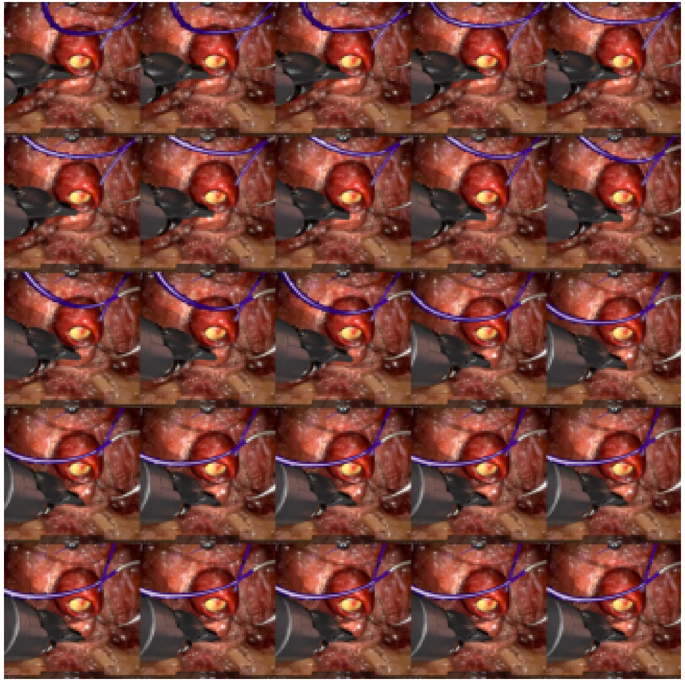

False Negative: Suturing
rarpexp002U2_28_C2_X4.npy
(data)

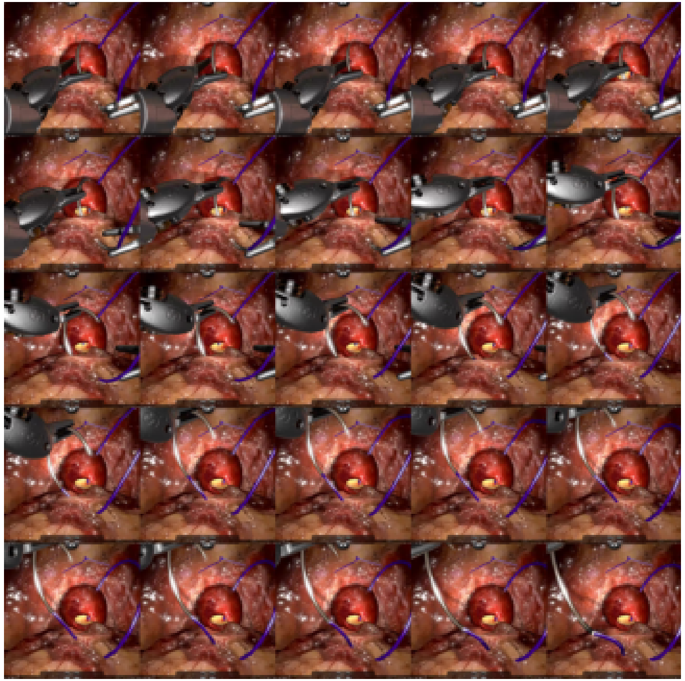

True Class Suturing

True Positive: Suturing
rarpexp002U2_3_C2_X4.npy
(data)

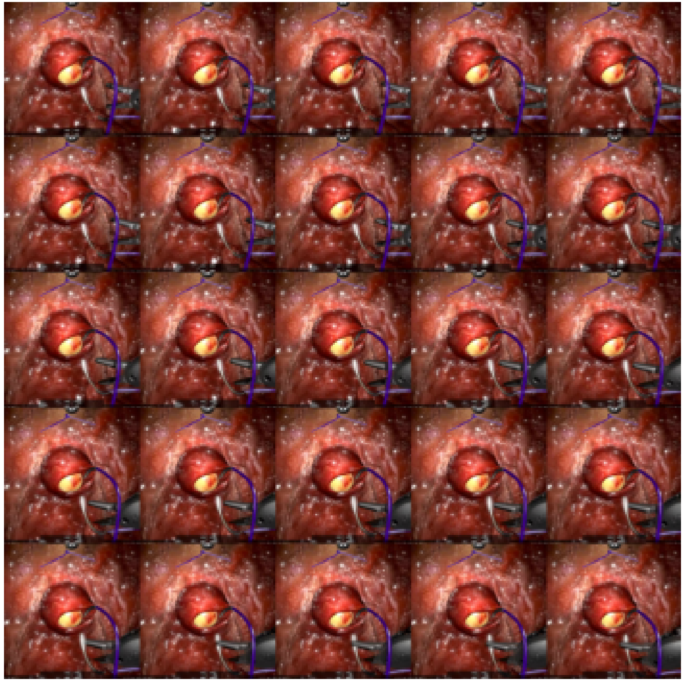

False Negative: Suturing
rarpexp002U2_2_C2_X4.npy
(data)

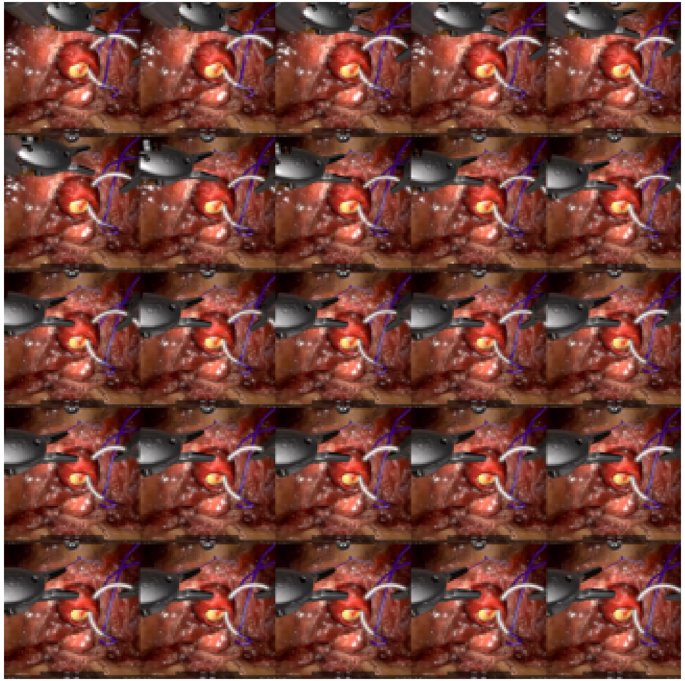

Supplement: Supplementary file 1 — Supplementary file1 (PDF 9969 KB) [file 11701_2025_2556_MOESM1_ESM.pdf]
